# Elevated Seawater Temperature and Infection with *Neoparamoeba perurans* Exacerbate Complex Gill Disease in Farmed Atlantic Salmon (*Salmo salar*) in British Columbia, Canada

**DOI:** 10.3390/microorganisms10051039

**Published:** 2022-05-17

**Authors:** Simon R. M. Jones, Derek Price

**Affiliations:** 1 Fisheries and Oceans Canada, Pacific Biological Station, Nanaimo, BC V9T 6N7, Canada; 2 Fisheries and Oceans Canada, Aquaculture Management, Courtenay, BC V9N 2M2, Canada; derek.price@dfo-mpo.gc.ca

**Keywords:** amoebic gill disease, complex gill disease, Atlantic salmon, aquaculture, temperature, salinity

## Abstract

Gill disorders and diseases are emergent health concerns affecting marine-farmed salmon, for which the causal factors are poorly understood in British Columbia (BC), Canada. This study sought to describe and compare spatial and temporal patterns of infection with *Neoparamoeba perurans*, the causal agent of amoebic gill disease, and visually assessed gill health scores in farmed Atlantic salmon. Gill tissue obtained during the Fisheries and Oceans Canada’s Fish Health Audit and Intelligence Program (DFO-FHAIP) between 2016 and 2020 were screened for *N. perurans* by qPCR. Semi-quantitative visual gill health assessments were conducted during the audits, and farms were assigned clinical AGD status based on microscopic visualization of *N. perurans* together with histopathological lesions. Seawater temperature and salinity data were collected from all active farms in the region during the study period. Trends in gill scores and associations with *N. perurans* infections were described and tested using an ordinal logistic mixed model. The amoeba was detected in 21% of 345 audited farms and in 12% of 1925 fish samples. Most (56%, n = 1898) samples had no visible gill damage (score = 0), and 23% had scores ≥ 2 (high). Distinct patterns of spatial and temporal variability in the rates of high gill scores and *N. perurans* infections are demonstrated. The model supported the statistically significant relationship observed between seawater temperature and the proportion of samples with elevated gill scores. The model also revealed a direct relationship between salinity and gill score but only in the presence of *N. perurans*. While the data suggest that histopathological lesions contributed to the gill scores, temperature and, to a lesser extent, salinity were significant risk factors of increased gill score. The results are discussed in the context of recently frequent thermal anomalies in the northeastern Pacific Ocean.

## 1. Introduction

Gill diseases are a health concern of marine-farmed Atlantic salmon (*Salmo salar*), and seven etiological types, including amoebic gill disease (AGD), have been recognised [1]. AGD is caused by infection with the dactylopodid amoeba *Neoparamoeba perurans* [2,3] and is considered emergent and economically important [4]. Treatments of the disease with freshwater or hydrogen peroxide baths are effective but costly, particularly in regions where the availability of freshwater is limited, and there is a need for specialised infrastructure and labour [5,6]. When left untreated, the disease causes mortality, reduced growth rates, and related production costs [7,8]. AGD is characterised by localised host responses, including epithelial edema, hyperplasia of the epithelial cells and mucous cells, fusion of lamellae, and the development of interlamellar vesicles [9]. In addition to the pathological lesions, the case definition includes an observation of amoebae in wet or histological preparations, in which at least one parasome, an intracellular symbiont of the amoeba, is observed [10,11].

The widespread global occurrence of AGD in cultured salmon [11,12,13,14] suggests a ubiquitous distribution of *N. perurans* in coastal waters [4]. Despite this, the amoeba has inconsistently been detected in samples of water, wild fish, or their ectoparasites, near to affected salmon aquaculture sites or elsewhere. Reservoirs of the infection are not known [4,15]. AGD was first detected in Washington State (WA, USA) in western North America in the mid-1980s [16]. The parasite has since been detected at a farm in Puget Sound, WA [17] with a history of AGD [18,19]. At that time, there was no evidence of AGD in 20 salmon collected from a farm on Vancouver Island, British Columbia (BC), Canada [17]. However, in 2013, *N. perurans* was first detected in farmed Atlantic salmon in BC, and in 2014, the parasite was detected at three farms, two of which were in association with gill pathology and elevated mortality [20]. More recent information suggests a broadening of the geographic ranges in BC both of *N. perurans* and AGD [21]. 

Coincident with the emergence of *N. perurans* and AGD among farmed salmon in BC, there have been anecdotal reports of sporadic to widespread gill diseases, including necrotic and erosive lesions [22], not unlike complex gill disease (CGD), as described elsewhere [1,23]. The extent to which the CGD-like lesions are associated with the presence of *N. perurans* is not known. This study reports on spatial, temporal, and seasonal patterns of *N. perurans* infection data and non-specific gross gill damage data from samples obtained through the Fish Health Audit and Intelligence Program of Fisheries and Oceans Canada (DFO-FHAIP). In addition, associations between the presence of the amoeba and the occurrence of elevated gill scores in farmed Atlantic salmon (*Salmo salar*) in BC were investigated in the context of farm-origin environmental data.

## 2. Material and Methods

### 2.1. Sample Collection and Gill Scoring

Visual assessments of gill health were conducted during necropsies of moribund and recently dead salmon during DFO-FHAIP audits, and of apparently healthy fish during sea lice audits. For the latter, salmon were collected by dip net from a box-seined subset of the population, anaesthetized in tricaine methane sulphonate, and released to the pen following examination. For moribund and recently dead specimens, the operculum was removed, gill arches were examined, and the type and extent of gross damage were noted and scored (Table 1). Filaments and arches were collected from the mid-line of the 2nd gill arch and preserved in 10% neutral-buffered formalin for histopathological examination. Filaments from the ventral margin of the 4th gill arch were collected and preserved in RNA later for molecular analyses. 

Seawater temperature and salinity data collected at 5 m depth from all active farms during the study period were provided by industry.

### 2.2. Histology

NBF-fixed samples were transferred to 70% ethanol for storage, dehydrated in an alcohol gradient, cleared in xylene, embedded with paraffin, and sectioned at 3–5 μm. Sections were mounted onto glass slides, stained with haematoxylin and eosin stains, cover-slipped, and examined using a compound microscope. Histopathological assessments were conducted at the Animal Health Centre, Abbotsford, BC, Canada. 

### 2.3. Neoparamoeba Perurans Quantitative PCR (qPCR)

DNA was extracted from approximately 20 mg of preserved gill tissue using the DNeasy 96 Blood and Tissue Extraction Kit (Qiagen, Hilden, Germany) and assessed for quantity and purity by spectrophotometry (Tecan, Männedorf, Switzerland). Parasite-specific DNA was quantified by qPCR in which 25 µL reactions consisted of 0.3 µM each of qPeruF (5′-GTTCTTTCGGGAGCTGGGAG-3′) and qPeruR (5′-GAACTATCGCCGGCACAAAAG-3′), 0.15 µM of NP Probe (6-FAM-CAATGCCATTCTTTTCGGA-MGBFNQ), 1× TaqMan Universal PCR Master Mix (Thermofisher, Burnaby, BC, Canada), 2 µL of DNA template, and RNase/DNase-free water [24]. The reactions were run in triplicate using a StepOne Plus real-time detection system (Applied Biosystems, Burnaby, BC, Canada). The number of copies per reaction (c/rxn) was estimated from a standard curve derived from the amplifications of a 10-fold serially diluted (10^1^ to 10^7^ c/rxn) double-stranded DNA fragment which included the *N. perurans* primer and probe binding sites (gBLOCK, IDT Technologies, Coralville, IA, USA). The limit of detection (LOD) for the qPCR assay was determined from a series of the gBLOCK fragments 2-fold serially diluted from 10^3^ c/rxn. The mean c/rxn, standard deviation, and coefficient of variation were calculated from 10 replicate qPCR reactions for each dilution. The limit of detection (LOD) was defined as the dilution at which gBLOCK DNA was first detected in 5 or more out of 10 replicate reactions and estimated to be 9.66 c/rxn (cycle threshold (*C*t) = 36.93). Samples were considered positive for *N. perurans* when *C*t ≤ LOD. 

### 2.4. Statistical Analysis

Individual qPCR data were cross-referenced with the DFO-FHAIP database and industry-provided environmental data. Farm- and audit-level data were extracted together with individual necropsy gill scores and histological diagnoses, and descriptive statistics were used to describe the trends.

An ordinal logistic mixed model was developed and used to estimate the probability of observing each level of gill score damage, as defined in Table 1, given spatial (fish health zone, FHZ: Aquaculture maps|Pacific Region (dfo-mpo.gc.ca)), temporal (year, season), and environmental factors (salinity, temperature), as well as the presence or absence of *N. perurans* and other fish-level factors. For this model, predictors were chosen based on unconditional statistical associations and guided by plausible biological relationships. The final model was chosen using AIC, and significance was set to *p* < 0.05. Spatial and temporal correlation was accounted for by including random effects for the FHZ and audit event. To aid in the convergence of the model, continuous predictors were scaled and centred. The models were fitted using the “ordinal” package [25] in R 4.1.2 [26].

## 3. Results

### 3.1. Temperature and Salinity

The mean seawater temperature and salinity (5 m depth) displayed limited inter-annual variation (Table S1). In contrast, mean temperature ranged from 8.0 °C to 13.7 °C among quarters and from 9.3 °C to 12.7 °C among fish health zones (FHZ, Table S1). The mean salinity varied little (28.0–28.3 ppt) among quarters and from 25.6 to 31.7 ppt among FHZs (Table S1). 

### 3.2. Neoparamoeba perurans in Fish Health Audits and Individual Fish 

Between 2016 and 2020, fish health audits were conducted at 345 Atlantic salmon production sites (Table S2). Approximately 21% of all audits were conducted in 2016 and 2017, and these accounted for roughly 6% of all fish examined. The parasite DNA was detected at 21.2% (n = 73) of audited farms, ranging from 8.3% of audits (n = 24) in 2016 to 34.0% (n = 106) in 2019. Overall, *N. perurans* was detected in all quarters (Q), ranging from 4.8% (n = 63) in Q2 (April–June) to 29.9% (n = 107) in Q4 (October–December). With the exception of FHZ 3.1 (n = 22 audits), the parasite was detected in all zones, ranging from 3.6% of audits (n = 83) in FHZ 3.3 to 50.0% (n = 48) in FHZ 3.4. The mean *C*t (±95% CI) value of positive samples was 32.7 ± 0.49.

**Figure 1:**
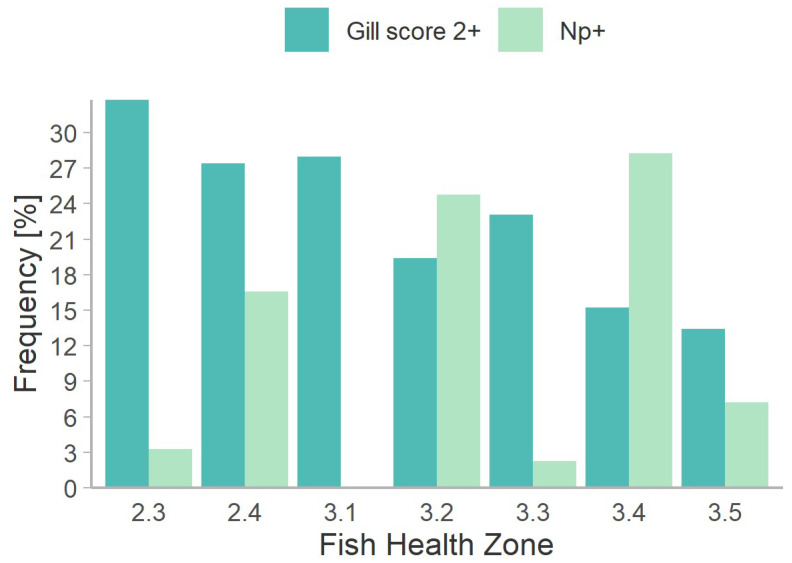
Proportions of farmed Atlantic salmon with visual gill scores ≥ 2 (n = 1898) or qPCR-detected *Neoparamoeba perurans* (n = 1925), from DFO-FHAIP data in British Columbia. All data summarised by health management zone from 2016 to 2020.

The parasite was detected by qPCR in 239 of 1925 (12.4%) Atlantic salmon examined during the 345 audits (Table S3). Overall, *N. perurans* was detected in all years, with prevalence increasing from 4.7% (n = 43) in 2016 to 17.2% (n = 709) in 2019, and declining to 14.2% (n = 472) in 2020 (*p* < 0.001, Chi^2^ = 38.28). Similarly, the parasite was detected in all seasons, ranging from 1.1% (n = 376) in Q2 to 21.0% (n = 576) in Q4 (*p* < 0.001, Chi^2^ = 89.2). The proportion of *N. perurans*-positive samples varied by zone (Figure 1). There was no evidence of the infection in FHZ 3.1 (n = 125), and among the other zones, prevalence ranged from 2.3% (n = 478) in FHZ 3.3 to 28.6% (n = 283) in FHZ 3.4 (*p* < 0.001, Chi^2^ = 206.6). 

### 3.3. Amoebic Gill Disease and Gill Damage

Clinical amoebic gill disease (AGD) was diagnosed at the farm level in three audits, all in 2018: one in Q3 in FHZ 3.3, and two in Q4 in FHZs 3.2 and 3.4. AGD was diagnosed in 10 of 47 salmon (21.3%) examined from FHZ 3.2 in Q4, in 5 of 48 salmon (10.4%) from FHZ 3.3 in Q3, and in 8 of 23 salmon (34.8%) from FHZ 3.4 in Q4. 

**Figure 2:**
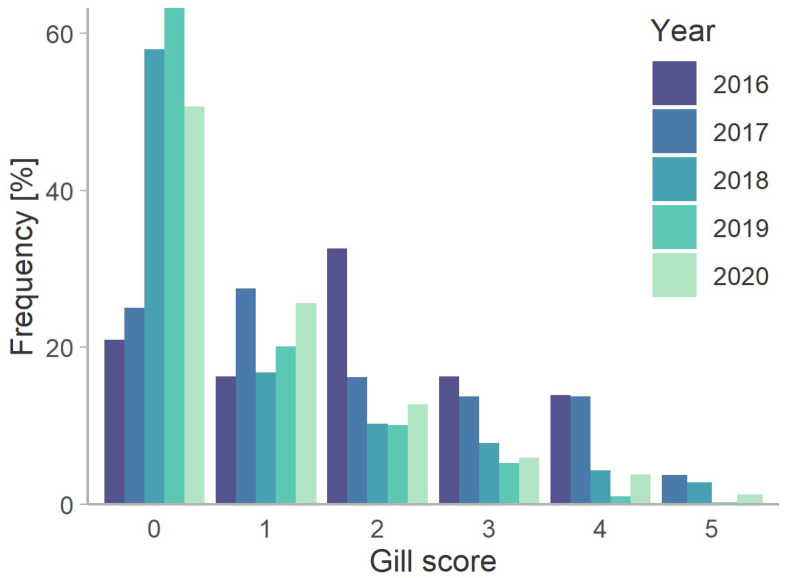
Atlantic salmon visual gill scores among all health management zones by year (n = 1898). Fisheries and Oceans Canada’s Fish Health Audit and Surveillance (DFO-FHAIP) data collected in British Columbia.

Overall, 56% of the 1898 salmon for which gills were scored had no visible gill damage (Figure 2). Between 2018 and 2020, the proportions of gills with scores between 1 and 5 ranged from 20.7% to 1.3%, respectively (Table S3; Figure 2). However, in 2016 and 2017, sampling was targeted towards suspected gill-disease cases resulting in the similar frequency distribution among gill scores (Figure 2). All samples were binned into those with scores <2 (low) and those with scores ≥ 2 (high). A total of 472 fish (23.6%) had high scores, and the rates of these ranged from 16.4% in 2019 to 62.8% in 2016 (*p* < 0.001, Chi^2^ = 84.07; Table 2). The rates of high scores among seasons were Q1 = 21.1%, Q2 = 17.3%, Q3 = 27.2% and Q4 = 25.1% (*p* = 0.002, Chi^2^ = 14.72). Variations among zones in the rates of high scores and the proportion of samples testing qPCR positive for *N. perurans* are shown in Figure 1 (*p* < 0.001, Chi^2^ = 39.71). Linear regression revealed a highly significant relationship between mean temperatures among zones and rates of high scores (*p* < 0.001), but not between mean temperatures and rates of *P. perurans* (*p* = 0.260). In contrast, mean salinities among zones regressed significantly with rates of high scores (*p* = 0.038) and rates of *P. perurans* (*p* = 0.022). The proportions of samples with histopathological lesions (lamellar hyperplasia and/or lamella fusion, lamellar telangiectasis, branchial stomatitis, thrombosis, filamental branchitis, and the presence of *Paramoeba* sp.) were consistently greater in samples with high versus low scores (Table 2). 

### 3.4. Days at Sea

Days-at-sea (DAS) data from 679 salmon samples ranged from 17 to 710 days. Of these, gill tissue from 46 tested positive for *N. perurans*. For sample sizes >100, the highest proportion of infections and lowest *C*t values, corresponding to highest concentrations of parasite-specific DNA, occurred between 200 and 399 DAS (Table 3). In contrast, mean gill scores were greatest between 400 and 599 DAS, coinciding with the highest proportions of samples with high gill scores (Table 3).

### 3.5. Logistic Model

A total of 1855 observations grouped into 337 audit events were included in the final model. A random term for FHZ was discarded in later iterations because all variance from this term was explained when salinity and temperature were added to the model. Despite being highly significant, a fixed term for days at sea (DAS) was not included in the model because the 36% of observations with DAS data were over-represented by Q2 (spring). The final model included fixed terms for *N. perurans* (presence/absence) salinity (ppt), temperature (°C), and the interaction between *N. perurans* and salinity, as well as a random term for audit event (Table 4).

**Figure 3:**
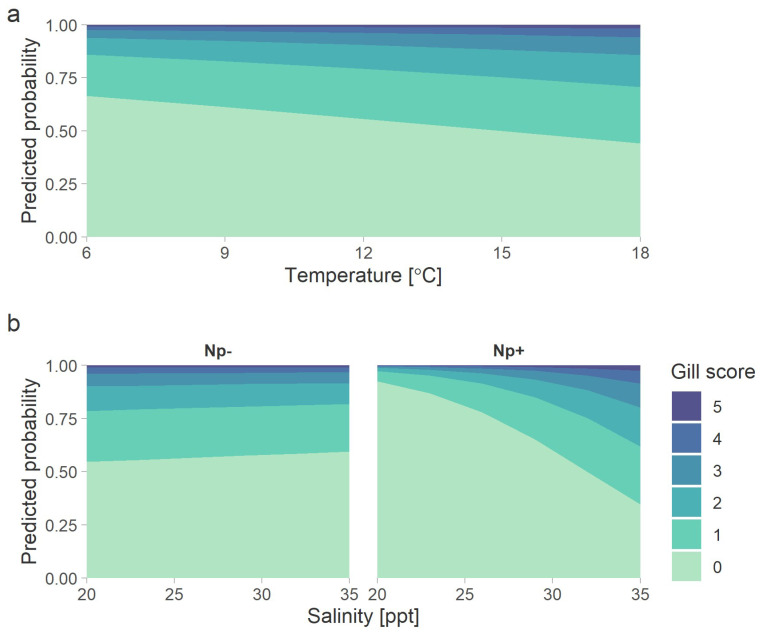
The effects of (**a**) temperature and (**b**) salinity in the absence (Np-) or presence (Np+) of *Neoparamoeba perurans* on the probability of gross gill scores, as predicted by the ordinal logistic model. See Table 1 for gill score criteria.

The model predicted that audits conducted at higher water temperatures were more likely to include samples with higher levels of gross gill damage (Figure 3a). For example, at 8 °C, 37% of gills were predicted to have some degree of damage, whereas at 15 °C, the predicted proportion with gill damage increased to 50%. The model also predicted that gill damage increased with salinity, but only in the presence of *N. perurans* (Figure 3b). In that case, at 25 ppt, approximately 20% of gills were predicted to have a score of 1 or greater, whereas at 30 ppt this proportion increased to 40%. Conversely, in the absence of the amoeba, predicted values were 44% and 42% of gills with some degree of damage at 25 and 30 ppt, respectively (Figure 3b).

## 4. Discussion and Conclusions

This comprehensive overview described and modelled pen-side visual gill scoring as an indicator of gill health generally, and in association with *Neoparamoeba perurans* infections and/or amoebic gill disease (AGD) in farmed Atlantic salmon (*Salmo salar*) in British Columbia (BC), Canada. Gills with no or negligible visible damage accounted for a majority of the samples examined, whereas gills with higher gill scores were increasingly rare. The pathological lesions observed in a relatively small proportion of the samples in each gill score category, including those without visible gill damage, were principally caused by hypertrophic or hyperplastic changes or by inflammation. However, the consistently higher frequencies with which these lesions occurred in gill samples with high versus low visible scores, combined with their relatively low overall prevalence, showed that the microscopic lesions contributed to, but did not entirely explain, the visible gill scores. Alternatively, infections with *N. perurans* were detected in the same samples at a prevalence nearly half that of the high scores. Gill scores and *N. perurans* infections displayed differential patterns of variability among fish health zones and over time. In fish health zone (FHZ) 3.1, nearly 40% of gills had high scores whereas *N. perurans* was not detected. Similarly, in FHZ 2.3, 33% of gills had high scores and the parasite was detected in only 3% of samples. In addition, the occurrence and severity of *N. perurans* infections peaked sooner than those of high gill scores following transfer of the salmon to seawater. Together, these observations indicate that the high gill scores were rarely explained by the presence of *N. perurans* infections. Similarly, with only three cases, clinical AGD was also not considered an explanatory factor of most gross or microscopic changes to the gill. Gill scores represent organ-wide gross changes caused by infectious or non-infectious insult and by the responses of gill tissues to these insults. The use of gill scores as a tool for assessing the need to treat AGD infections in farmed Atlantic salmon was shown initially to be most reliable in severe cases [9], and later formalised into a six-stage system [27]. Modifications of the latter system are now used in semi-quantitative assessments of the severities of other gill diseases [28,29]. Thus, while visible and histological changes were consistently observed in gill samples collected throughout the salmon aquaculture industry in BC between 2016 and 2020, their etiology remains poorly defined, reminiscent of complex gill disease (CGD). 

The causes of CGD and other proliferative gill diseases in marine-cultured salmon include infectious and non-infectious agents [1,23,30,31]. Confirmation of one or more infectious etiologies for CGD is limited by an absence of controlled laboratory infection models, and the extent to which specific agents are causal of or consequential to gill pathology remains unclear. Cnidaria, including pelagic stages or sessile stages which have been released into net pens through the action of in situ net-washing, have also been associated with CGD-like gill damage in farmed salmon [28,32,33,34,35,36,37,38]. However, more work is required to better define the ecological, environmental, and production factors which contribute to net-wash-effluent-induced gill pathology [39]. In the present study, the variation of gill scores among management zones and seasons suggests a significant relationship with seawater temperature. Consistent with this and regardless of the presence of *N. perurans*, the model unambiguously supports a positive association between temperature and the frequency of samples with visible gill damage. In contrast, the previously reported interaction of temperature and *N. perurans* on gill scores [40] may have resulted from the greater intensities of the laboratory infections than those measured in the present study. The more general recognition of elevated temperature as a risk factor for several gill diseases [23] is likely due to poorly documented effects on physiological processes in the host and pathogen. Nevertheless, rearing salmon in marine areas with elevated temperature is shown here to be a risk factor for visible gill damage in BC.

The severity of CGD-associated gill damage in marine pen-reared salmon tends to be seasonal, being greatest in the late summer and autumn [1,23]. While this pattern may be consistent with a temperature effect, the more general possibility that gill lesion severity increases with time, perhaps because of the cumulative effects of continual exposure to gill irritants [29], has received little scientific attention. In contrast to the reported accumulation of parasites with time at sea [41], the magnitude and longevity of gill damage in marine-reared salmon will depend on the nature of the infectious or non-infectious insult, and simultaneous or sequential exposures to multiple insults will complicate the outcome. The fact that gill pathology was elevated one day but not eight days following a single exposure to netwash effluent, in which evidence of the harmful hydroid *Ectopleura larynx* was detected [39], illustrates the transience of the gill response to certain insults. In the present study, there was an apparent tendency for the prevalence both of *N. perurans* and more severe gill lesions to increase with days at sea. Confirmation and an adequate interpretation of these findings however will require a more spatially and temporally balanced dataset.

Our poor understanding of an explanation for the apparent rapidity with which *N. perurans* has become established within marine-cultured salmon in BC since 2013, including Chinook salmon (*Oncorhynchus tshawytscha*; AGD was diagnosed in two audits in Q1 of 2019, FHZ 2.3 and 3.2, and in one audit in Q1 of 2020, FHZ 2.3; DFO-FHAIP unpublished data) reflects knowledge gaps in marine reservoirs and factors associated with risk of exposure to *N. perurans*. Although the protozoan occurs in association with benthic substrata, parasitic invertebrates, and net pen fouling communities [4,8,17,42,43,44], the extent with which these habitats consistently serve as sources of the amoeba throughout its range is uncertain. Similarly, the earlier conclusion that wild marine fishes are unlikely to be important reservoirs of the parasite [15,45] was confirmed in BC waters by surveillance of ocean-entry-year salmon between 2016 and 2021. In the latter study, *N. perurans* was detected by qPCR in 4 of 2365 chum salmon (*O. keta*), 0 of 1087 pink salmon (*O. gorbuscha*), 0 of 99 Chinook salmon, 0 of 22 sockeye salmon (*O. nerka*), and in 0 of 12 coho salmon (*O. kisutch*) (S. Jones, unpublished data). In the present study, although there was no obvious relationship between gill scores or histological lesions and infection with *N. perurans*, the ordinal model revealed a significant relationship between salinity and gill score in the presence of *N. perurans*. In this context, it is noteworthy that the parasite was not detected in FHZ 3.1, in which the mean salinity was lowest among zones. Thus, our observations may reflect a role of elevated salinity in promoting parasite viability, as reported elsewhere [4], despite the apparent rarity of temperature conditions in BC waters conducive to the development of AGD. However, recent occurrences of seawater thermal anomalies in the northeast Pacific Ocean [46,47] have coincided with episodes of increased surface temperatures in coastal zones, along with associated multitrophic ecological impacts [48,49,50]. Given the detection of *N. perurans* in all years, all seasons and virtually all health management zones, any warming of the oceanographic climate along Canada’s west coast, will increase the risk for the emergence of widespread AGD in addition to other proliferative gill diseases among marine-farmed salmon in BC.

In conclusion, distinct patterns of spatial and temporal variability in the rates of gill scores and *N. perurans* infections were demonstrated. While the data suggest that histopathological lesions contributed to, but did not entirely explain, the gill scores, temperature and, to a lesser extent, salinity were significant risk factors of increased gill score [41]. The characterisation of molecular biomarkers as indicators and/or predictors of CGD [29] will be helpful in improving our understanding of the underlying mechanisms of disease. 

## Figures and Tables

**Table 1:** Gross damage criteria used to assign a score to salmon gill.

Score	Filament Damage	Extent (%)
0	None visible	0
1	Limited thickening, or very few affected	<10
2	Frequent thickening of tips	10–25
3	Thickening on most tips, extending to ≤50% of length	25–50
4	Thickening on most, to more than 50% of length	50–75
5	Most or all thickened along entire length	75–100

**Table 2:** The effect of Atlantic salmon gill scores (low, <2; high, ≥2) on the number (percent) of samples with histopathological lesions between 2016 and 2020.

Score	N (%)	Pathology ^1^					
		GLH/GLF	PAP	GFB	GLT	BST	GTH
<2	1437 (76.4)	39 (2.7)	8 (0.6)	15 (1.0)	9 (0.6)	8 (0.6)	8 (0.6)
≥2	444 (23.6)	55 (12.4)	11 (2.5)	22 (5.0)	6 (1.4)	6 (1.4)	4 (0.9)

^1^ GLH/GLF, gill lamellar hyperplasia/fusion; PAP, *Paramoeba* sp.; GFB, gill filament branchitis; GLT, gill lamellar telangiectasis; BST, branchial stomatitis; GTH, gill thrombosis.

**Table 3:** The effect of days at sea on infection with *Neoparamoeba perurans* and mean gill score in farmed Atlantic salmon (*Salmo salar*) in British Columbia, Canada between 2016 and 2020 (see text).

Days at Sea	N_t_	*N. perurans*		Gill Score	
		N_p_	Mean *C*t	N ≥ 2	Mean
0–199	171	5 (2.9)	36.45	23 (13.4)	0.47
200–299	103	9 (8.7)	28.27	9 (8.7)	0.38
300–399	112	13 (11.6)	32.28	24 (21.4)	0.72
400–499	159	8 (5.0)	34.67	52 (32.7)	1.12
500–599	106	3 (2.3)	33.37	40 (37.7)	1.26
600–699	25	5 (20.0)	33.36	5 (16.0)	1.00
700–799	3	3 (100.0)	30.04	0	1.00

N_t_, number of fish examined; N_p_, number positive by qPCR; N ≥ 2, number with high gill scores. Percentages in parentheses.

**Table 4:** Coefficients, standard error, 95% confidence intervals, and *p*-values for the final ordinal logistic model.

Term	Estimate	Std. Error	95% Conf. Interval	*p* Value
Gill score thresholds				
0|1	0.31	0.10	0.12–0.50	
1|2	1.42	0.10	1.22–1.63	
2|3	2.34	0.12	2.11–2.56	
3|4	3.32	0.14	3.05–3.60	
4|5	4.63	0.21	4.21–5.04	
*N. perurans*	−0.27	0.25	−0.77–0.22	
Salinity (scaled)	−0.03	0.07	−0.16–0.09	
Temperature (scaled)	0.19	0.08	0.05–0.34	0.01
*N. perurans* × Salinity (scaled)	0.59	0.26	0.08–1.09	0.02

## Data Availability

The data presented in this study may be available on request from the corresponding author.

## Supplementary Materials

The following supporting information can be downloaded at: https://www.mdpi.com/article/10.3390/microorganisms10051039/s1. Table S1: Overall mean 5 m seawater temperature and salinity (range) by year, yearly quarter (Q) and fish health management zone from farm data in British Columbia between 2016 and 2020. Table S2: Number of DFO-FHAIP audits conducted (N_A) and number positive for *Neoparamoeba perurans* (N_P) by quantitative PCR in farmed Atlantic salmon (*Salmo salar*) in British Columbia, Canada: by year, quarter (Q) and fish health zone (Z). Table S3: Number of farmed Atlantic salmon (*Salmo salar*) examined during DFO-FHAIP audits (N_F), number positive for *Neoparamoeba perurans* (N_P) by quantitative PCR, and number with gill scores ≥ 2 (P) (see text): by year, quarter (Q) and fish health zone (Z), in British Columbia, Canada.
