# Supplementary material for: Elevated Seawater Temperature and Infection with Neoparamoeba perurans Exacerbate Complex Gill Disease in Farmed Atlantic Salmon (Salmo salar) in British Columbia, Canada
Source: Microorganisms. 2022 May 17;10(5):1039. doi: 10.3390/microorganisms10051039 (PMC9147833; doi:10.3390/microorganisms10051039)
Supplement: Supplementary file 1 [file microorganisms-10-01039-s001.zip › microorganisms-1715700-supplementary.pdf]

Supplementary Information

Table S1. Overall mean 5-meter seawater temperature and salinity (range) by year, yearly quarter (Q) and fish health management zone from farm data in British Columbia between 2016 and 2020.

	Temperature (°C)		Salinity (PSU)	
	N	Mean (range)	N	Mean (range)
Year				
2016	1556	11.2 (5.8 – 20.6)	1658	27.8 (16.2 – 35.0)
2017	1651	10.2 (4.8 – 20.8)	1651	28.0 (13.3 – 34.5)
2018	1719	10.8 (5.7 – 21.5)	1720	28.2 (16.4 – 35.9)
2019	1891	11.0 (6.2 – 20.8)	1893	28.9 (16.1 – 35.4)
2020	1716	10.4 (5.5 – 19.6)	1717	27.6 (10.6 – 34.1)
Quarter				
Q1	2509	8.0 (4.8 – 11.4)	2664	28.2 (10.6 – 35.1)
Q2	2731	11.3 (6.9 – 20.6)	2835	28.2 (18.3 – 35.4)
Q3	2436	13.7 (9.0 – 21.5)	2514	28.3 (13.3 – 35.9)
Q4	2386	10.0 (5.2 – 16.1)	2469	28.0 (13.6 – 35.2)
Zone				
2.3	2570	11.2 (6.9 – 17.5)	2570	27.3 (16.2 – 33.0)
2.4	2213	11.0 (5.4 – 18.9)	2257	29.1 (10.6 – 34.9)
3.1	1403	12.7 (4.8 – 21.5)	1403	25.6 (13.3 – 30.6)
3.2	1074	9.9 (6.8 – 17.4)	1153	29.1 (20.2 – 33.3)
3.3	1977	9.6 (6.2 – 16.7)	2115	28.4 (17.4 – 34.0)
3.4	622	9.3 (7.0 – 12.6)	711	31.7 (24.3 – 35.9)
3.5	203	9.3 (6.2 – 13.9)	273	29.1 (24.4 – 32.9)

Table S2. Number of DFO-FHAIP audits conducted (N_A) and number positive for *Neoparamoeba perurans* (N_P) by quantitative PCR in farmed Atlantic salmon (*Salmo salar*) in British Columbia, Canada: by year, quarter (Q) and Fish Health Zone (Z).

Q[1]	Z[2]	Year									
		2016		2017		2018		2019		2020	
		N_A	N_P	N_A	N_P	N_A	N_P	N_A	N_P	N_A	N_P
1	2.3	--[3]		3	0	5	0	4	4	6	1
	2.4	--		1	0	5	0	6	3	6	0
	3.1	--		2	0	3	0	--		1	0
	3.2	--		--		--		4	0	4	1
	3.3	--		--		--		7	0	4	0
	3.4	--		2	1	2	1	4	2	2	0
	3.5	--		--		--		1	1	2	0
2	2.3	--		3	0	5	0	0	0	1	0
	2.4	--		1	0	4	0	6	0	--	
	3.1	--		--		3	0	2	0	--	
	3.2	--		2	0	4	0	3	0	--	
	3.3	--		5	0	5	0	8	0	--	
	3.4	--		1	1	4	1	4	1	--	
	3.5	--		--		1	0	1	0	--	
3	2.3	4	0	1	0	4	0	4	0	3	0
	2.4	1	0	3	0	2	0	4	4	2	2
	3.1	--		--		3	0	3	0	3	0
	3.2	1	0	2	0	4	1	4	3	6	2
	3.3	2	0	5	0	7	0	7	1	6	1
	3.4	1	0	1	1	5	2	4	2	4	2
	3.5	1	0	--		1	0	2	1	1	0
4	2.3	4	0	1	0	3	0	5	1	--	
	2.4	1	0	5	1	6	1	6	5	6	3
	3.1	--		--		1	0	1	0	--	
	3.2	2	1	2	0	7	3	4	3	5	2
	3.3	5	0	4	0	6	0	7	1	5	0
	3.4	1	1	3	1	3	3	3	2	4	3
	3.5	1	0	--		2	0	2	1	2	0

[1]Quarter: 1 (January – March), 2 (April – June), 3 (July – September), 4 (October – December).

[2]Fish Health Zone: see text.

[3] -- no audits conducted.

Table S3. Number of farmed Atlantic salmon (*Salmo salar*) examined during DFO-FHAIP audits (N_F), number positive for *Neoparamoeba perurans* (N_P) by quantitative PCR, and number with gill scores ≥ 2 (P) (see text): by year, quarter (Q) and Fish Health Zone (Z), in British Columbia, Canada.

Q[1]	Z[2]	Year														
		2016			2017			2018			2019			2020		
		N_F	N_P	P	N_F	N_P	P	N_F	N_P	P	N_F	N_P	P	N_F	N_P	P
1	2.3	--[3]			3	0	2	19	0	17	33	7	2	47	1	18
	2.4	--			1	0	0	11	0	10	45	12	6	32	0	9
	3.1	--			3	0	1	4	0	4	--			5	0	0
	3.2	--			--			--			19	0	1	28	4	1
	3.3	--			--			--			46	0	4	19	0	1
	3.4	--			2	1	0	4	1	2	22	5	0	14	0	2
	3.5	--			--			--			8	2	0	14	0	0
2	2.3	--			4	0	3	40	0	12	--			8	0	1
	2.4	--			1	0	0	30	0	14	45	0	7	--		
	3.1	--			--			23	0	10	12	0	0	--		
	3.2	--			3	0	2	24	0	2	12	0	1	--		
	3.3	--			8	0	1	37	0	6	54	0	5	--		
	3.4	--			2	1	0	28	1	1	30	2	1	--		
	3.5	--			--			7	0	0	8	0	0	--		
3	2.3	7	0	4	1	0	0	31	0	13	27	0	9	23	0	15
	2.4	3	0	1	7	0	6	9	0	1	26	13	2	4	2	3
	3.1	--			--			28	0	6	15	0	5	14	0	8
	3.2	1	0	1	3	0	0	21	2	4	29	11	7	47	15	12
	3.3	5	0	5	11	0	4	48[4]	0	11	53	1	5	41	1	8
	3.4	1	0	0	1	1	1	42	9	7	30	8	7	32	15	9
	3.5	2	0	2	--			10	0	0	7	2	1	1	0	1
4	2.3	5	0	2	1	0	0	23	0	2	33	2	0	--		
	2.4	1	0	1	12	1	10	37	1	1	37	19	16	31	7	4
	3.1	--			--			7	0	1	14	0	0	--		
	3.2	2	1	1	5	0	4	47[4]	15	4	23	19	9	35	8	7
	3.3	13	0	9	7	0	3	41	0	14	58	9	24	43	0	8
	3.4	2	1	1	5	1	1	23[4]	13	5	18	8	3	27	14	3
	3.5	1	0	0	--			13	0	5	7	3	2	13	0	0

[1]Quarter: 1 (January – March), 2 (April – June), 3 (July – September), 4 (October – December).
[2]Zone: see Figure 1 (map).
[3] -- no audits conducted.
[4]Clinical amoebic gill disease diagnosed (see text)
